# An ErbB Lineage Co-Regulon Harbors Potentially Co-Druggable Targets for Multimodal Precision Therapy in Head and Neck Squamous Cell Carcinoma

**DOI:** 10.3390/ijms232113497

**Published:** 2022-11-04

**Authors:** Markus Bredel, Hyunsoo Kim, James A. Bonner

**Affiliations:** 1 Department of Radiation Oncology, O’Neal Comprehensive Cancer Center, Heersink School of Medicine, The University of Alabama at Birmingham, Birmingham, AL 35294, USA; 2 Lineberger Comprehensive Cancer Center, University of Northern Carolina at Chapel Hill, Chapel Hill, NC 27599, USA

**Keywords:** glioblastoma, ErbB, regulon, multimodal precision therapy, co-druggable targets, head and neck squamous cell carcinoma

## Abstract

The ErbB lineage of oncogenic receptor tyrosine kinases is frequently overexpressed in head and neck squamous cell carcinomas. A common co-regulon triggered by the ErbB proteins; involving shared signaling circuitries; may harbor co-druggable targets or response biomarkers for potential future multimodal precision therapy in ErbB-driven head and neck squamous cell carcinoma. We here present a cohort-based; genome-wide analysis of 488 head and neck squamous cell carcinomas curated as part of The Cancer Genome Atlas Project to characterize genes that are significantly positively co-regulated with the four ErbB proteins and those that are shared among all ErbBs denoting a common ErbB co-regulon. Significant positive gene correlations involved hundreds of genes that were co-expressed with the four ErbB family members (*q* < 0.05). A common; overlapping co-regulon consisted of a core set of 268 genes that were uniformly co-regulated with all four ErbB genes and highly enriched for functions in chromatin organization and histone modifications. This high-priority set of genes contained ten putative antineoplastic drug-gene interactions. The nature and directionality of these ten drug-gene associations was an inhibiting interaction for seven (PIK3CB; PIK3C2B; HDAC4; FRK; PRKCE; EPHA4; and DYRK1A) of them in which the drug decreases the biological activity or expression of the gene target. For three (CHD4; ARID1A; and PBRM1) of the associations; the directionality of the interaction was such that the gene predicted sensitivit y to the drug suggesting utility as potential response biomarkers. Drug-gene interactions that predicted the gene product to be reduced by the drug included a variety of potential targeted molecular agent classes. This unbiased genome-wide analysis identified a target-rich environment for multimodal therapeutic approaches in tumors that are putatively ErbB-driven. The results of this study require preclinical validation before ultimately devising lines of combinatorial treatment strategies for ErbB-dependent head and neck squamous cell carcinomas that incorporate these findings.

## 1. Introduction

The ErbB lineage of family proteins consists of four members (ErbB1-4) that share significant structural homology and function as homo- or heterodimeric receptor tyrosine kinases (RTK) to activate downstream transforming pathway signaling [1,2,3,4,5,6]. ErbB proteins exist in an equilibrium between a tethered, inactive conformation and an extended, active state poised toward the formation of homo- or heterodimers with each other, normally in a ligand-dependent manner [7]. Ligands can either display receptor specificity or bind to one or more related ErbB receptors [1]. ErbB2 lacks a known ligand but has a structure that resembles a ligand-activated state and favors dimerization [8]. It readily forms heterodimers with the other family members [8]. Regulation of ErbBs, which includes dense feedback and feedforward loops, and crosstalk, leads to a massive rearrangement of gene regulation and expression patterns that drive oncogenic processes [9,10,11]. Multi-level transcriptional cascades initiated by ErbBs have been identified [9,10,11].

Head and neck squamous cell carcinomas are complex diseases that demonstrate a remarkable multiplicity of genetic or epigenetic events [12,13], many of which converge into ErbB signaling circuitries [14].Simultaneous overexpression of multiple ErbB receptors in most head and neck squamous cell carcinoma cases suggests recurrent involvement of receptor heterodimers [15].The identification of the molecular importance of ErbBs in head and neck squamous cell carcinomas prompted evaluation of their potential as therapeutic targets [12,16,17]. ErbB1, or Epidermal Growth Factor Receptor (EGFR), is consistently overexpressed and occasionally mutated and amplified in head and neck squamous cell carcinomas [13,18,19] and is an established target of monoclonal antibody (mAb) therapies with a meaningful clinical impact [12]. Elevated HER2 expression has been reportedly associated with worse prognosis, increased recurrence, and decreased survival in head and neck squamous cell carcinoma and is considered a therapeutic target [20,21]. ErbB3 has been associated with decreased survival in head and neck squamous cell carcinoma [22], and an anti-ErbB3 mAb has shown promising initial clinical activity [23]. Although ErbB4 is not a prognostic parameter for survival outcomes in head and neck squamous cell carcinomas, it is highly overexpressed and thus a potential target for molecular therapies pending further investigation [8,15].

Nonetheless, the network context of a molecular target can affect the efficacy of therapies that target its protein. The molecular complexity of head and neck squamous cell carcinomas helps explain why there are clinical settings in which targeting a single protein, such as an individual ErbB receptor, can fail. For example, targeting EGFR has been shown to have a limited clinical effect in situations of primary or acquired EGFR inhibitor resistance despite its biologic prominence [24]. This paradox is resolved by the fact that the deregulation of an oncoprotein in head and neck squamous cell carcinoma does not exist in isolation. Rather, the functional organization of ErbB family members in a complex functional network of interactions predicts that mono-therapeutic approaches can have limited clinical efficacy in some situations. We, therefore, hypothesized that the characterization of a common ErbB co-regulon might identify co-regulated and potentially co-druggable targets or response biomarkers for future multimodal precision therapy in head and neck squamous cell carcinoma. We explored existing molecular databases to assess this premise.

## 2. Methods and Results

**Figure 1:**
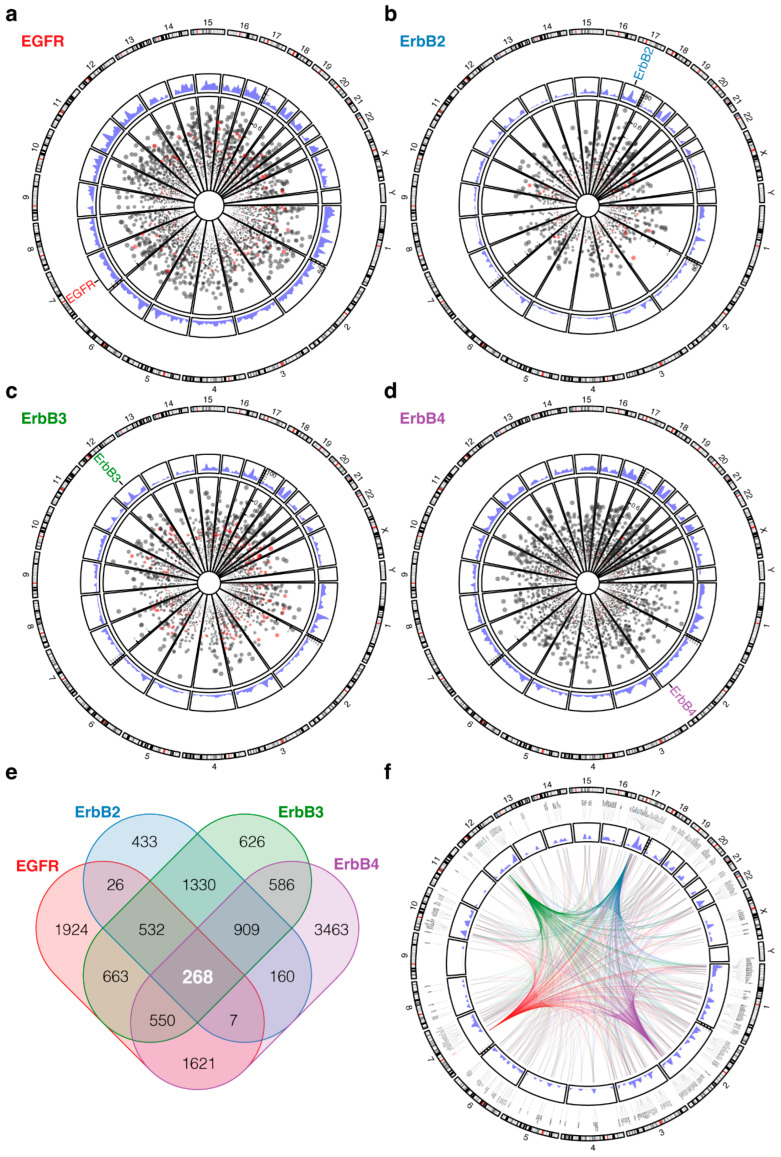
ErbB lineage co-regulon in head and neck squamous cell carcinoma. (**a**–**d**) Circular genome (CIRCOS) visualization of genes that are significantly positively co-regulated with the individual ErbB lineage family genes EGFR (**a**), ErbB2 (**b**), ErbB3 (**c**), and ErbB4 (**d**) in 488 head and neck squamous cell carcinomas. Numbers in the CIRCOS structure indicate chromosomes. Dots indicate individual genes. Gene positions from the center of the CIRCOS are proportional to the Spearman’s rank correlation coefficient. Dot size corresponds to false-discovery rate estimated q-value with larger size representing a smaller (more significant) q-value. Blue peaks denote the number of significantly co-expressed genes according to cytoband/chromosome position. (**e**) Euler diagram depicting the relationship and overlap of sets of genes that are significantly co-expressed with each of the four ErbB family genes. A core set of 268 genes was uniformly co-regulated with all four ErbBs. (**f**) CIRCOS structure visualizing the genome position of a core set of 268 genes that are significantly co-regulated with all four ErbBs thus denoting a common ErbB lineage co-regulon.

We employed an unbiased genome-wide approach in 488 head and neck squamous cell carcinoma patients curated in The Cancer Genome Atlas to identify genes that are significantly positively co-expressed with the four ErbB proteins. Gene correlations were derived from batch-normalized RNA sequencing (RNAseq) data using Spearman’s rank correlation coefficient, and significant correlations were determined by false-discovery rate estimated q-values. As displayed on circular genomes, this analysis revealed hundreds of genes that are significantly (*q* < 0.05) co-expressed with each of the ErbBs (Figure 1a–d and Supplement). Those genes mapped across the human genome without a predilection for distinct genomic loci (Figure 1a–d).

**Figure 2:**
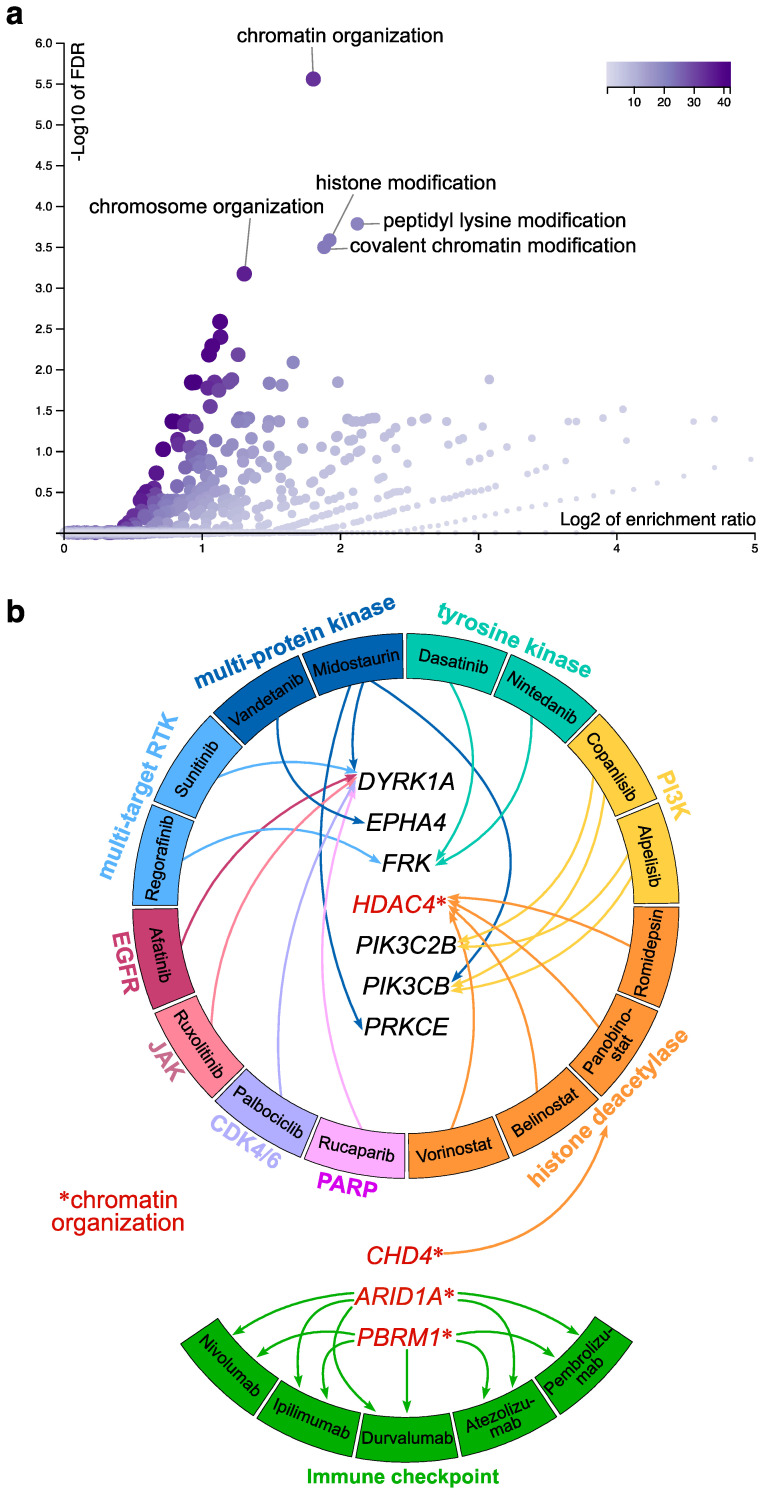
Functional enrichment and druggability of an ErbB lineage co-regulon in head and neck squamous cell carcinoma. (**a**) Over-representation analysis of enrichment of a uniform ErbB lineage co-regulon, consisting of a core set of 268 genes, for specific molecular functions. Dots represent individual molecular functions positioned according to their false-discovery rate and enrichment ratio. Significant enrichment of the co-regulon for functions in chromatin/chromosome organization and histone modifications. (**b**) Annotation of the ErbB co-regulon with respect to known drug-gene interactions involving approved antineoplastic drugs, using the Drug Gene Interaction Database (DGIdb). The co-regulon contained ten putative antineoplastic drug-gene interactions. The nature and directionality of these ten drug-gene associations was an inhibiting interaction for seven (PIK3CB, PIK3C2B, HDAC4, FRK, PRKCE, EPHA4, and DYRK1A) of them in which the drug decreases the biological activity or expression of the gene target. For three (CHD4, ARID1A, and PBRM1) of the associations, the directionality of the interaction was such that the gene predicted sensitivity to the drug suggesting utility as potential response biomarkers. Drug-gene interactions that predicted the gene product to be reduced by the drug included a variety of potential targeted molecular agent classes.

Given substantial redundancy of signaling circuitries involving the ErbBs and the documented signaling potency emanating from ErbB receptor combinations [25,26,27,28,29,30], we then investigated the relevant genes for a common ErbB co-regulon as a high-priority set of genes to be further analyzed for druggable targets or biomarkers. We determined an overlapping set of 268 genes that were consistently co-expressed with all ErbB family members (Figure 1e,f and Supplement). Strikingly, this set of core genes was highly enriched for functions in chromatin organization and histone modifications, as modeled by over-representation analysis using R package WebGestalt [31] (Figure 2a). Many studies show epigenetic regulation, including chromatin remodeling and histone post-translational covalent modifications, has a critical role in the formation and progression of head and neck squamous cell carcinomas [32]. We finally analyzed this gene set for potential druggability with an aim to identify targetable areas for multimodal treatment strategies. We used the Drug Gene Interaction Database (DGIdb [33]) to annotate our genes of interest with respect to known drug-gene interactions and potential druggability, with an emphasis on approved antineoplastic drugs. This analysis identified ten putative drug-gene interactions, four of which were involved in chromatin organization (Figure 2b). The nature and directionality of these ten drug-gene associations was an inhibiting interaction for seven (PIK3CB, PIK3C2B, HDAC4, FRK, PRKCE, EPHA4, and DYRK1A) of them in which the drug decreases the biological activity or expression of the gene target (Figure 2b). For three (CHD4, ARID1A, and PBRM1) of the associations, the directionality of the interaction was such that the gene predicted sensitivity to the drug suggesting utility as potential response biomarkers (Figure 2b). Drug-gene interactions that predicted the gene product to be reduced by the drug included a variety of potential targeted molecular agent classes (Figure 2b).

## 3. Discussion

Herein, we utilized a systematic, genome-wide approach to characterize an ErbB co-regulon in head and neck squamous cell carcinomas with the goal of defining drug-gene associations that may represent action points for tumors that are putatively driven by the co-regulon. We found this co-regulon is highly enriched for functions in chromatin remodeling and histone modifications and harbors potentially actionable, co-druggable targets and response biomarkers for future investigations of multimodal precision therapy. These findings can serve as direction for preclinical investigations followed by clinical investigations of promising results.

DYRK1A, a serine/threonine kinase that belongs to the dual-specificity tyrosine phosphorylation-regulated kinase (DYRK) family, has an emerging role in cancer biology through its ability to regulate cell cycle progression, DNA damage repair, transcription, ubiquitination, tyrosine kinase activity, and cancer stem cell maintenance [34]. It has been shown that DYRK1A is required for the maintenance of cancer stemness, contributing to tumorigenic potential in oral/oropharyngeal squamous cell carcinoma [35]. Given its multifaceted role in various cancer-related biological processes, there has been significant interest in DYRK1A as a potential therapeutic target [34]. Multi-target RTK, multiprotein kinase, tyrosine kinase, JAK, CDK4/6, and PARP inhibitors all bind to DYRK1A (Figure 2b).

Ephrin receptor A4 (EPHA4) regulates many normal biologic and pathologic processes. Evidence suggests a role of EPHA4 in tumor development and progression [36,37]. Elevated expression of EPHA4 was found in head and neck cancers [38], although differential outcomes of EphB4-ephrinB2 (its ligand) signaling have been reported in these neoplasms. The receptor has been considered an attractive target for cancer therapy [37,39]. Our DGIdb search identified the multiprotein kinase inhibitor Vandetanib among drugs that target EPHA4 (Figure 2b).

Fyn related Src family tyrosine kinase (FRK) is a non-receptor tyrosine-protein kinase whose role in cancer remains controversial. While originally attributed a potential tumor-suppressive function, in recent years, further functional characterization revealed that FRK might potentially play an oncogenic role [40]. As such, it has been shown to be oncogenic in lung cancer cells by enhancing stemness [41]. Little is known about FRK in head and neck squamous cell carcinomas. Multi-target RTKs and tyrosine kinase inhibitors target FRK (Figure 2b).

Histone deacetylase 4 (HDAC4) is responsible for the deacetylation of lysine residues on the N-terminal aspect of core histones, thereby providing an epigenetic tag that represses transcription. HDACs also deacetylate non-histone cellular substrates and influence a variety of biological processes that govern cancer initiation and progression [42]. HDAC4 is upregulated in head and neck cancer tissues [43]. HDAC4 is targeted by HDAC inhibitors (Figure 2b). This class of agents has an emerging role in cancer therapy and demonstrated synergism when combined with other cancer drugs [44,45]. HDAC inhibitors have been used to target and degrade HDAC4 and disrupt cancer stem cells in head and neck cancers [43,46]. They have also demonstrated synergistic antitumor effects when combined with EGFR inhibitors [47,48], suggesting a promising combinatorial therapeutic route. Part of this synergism could be due to histone acetylation-independent blockade of the EGFR axis [48,49] or reversal of epithelial-to-mesenchymal transition associated EGFR inhibitor resistance [47].

Dysregulation of phosphatidylinositol 3-kinase (PI3K) signaling has been a molecular target in both human papilloma virus (HPV)-positive and HPV-negative head and neck squamous cell carcinomas as it is the most frequently altered oncogenic pathway in these neoplasms, in part brought about by PI3KCA gain-of-function mutations [13,50,51,52,53,54,55]. PI3K pathway mutations may serve as predictive biomarkers for treatment selection in head and neck cancers [56,57]. PIK3CB encodes an isoform of the catalytic subunit of PI3K, namely the catalytic subunit for PI3Kbeta (PI3KB). PI3-kinases are responsible for coordinating a diverse range of cell functions such as proliferation, survival, migration, and oncogenic transformation [58,59]. Somatic mutations in PIK3CB have been found in diverse cancer lineages [60,61]. Evidence suggests that PIK3CB is responsible for driving tumorigenesis in the absence of mutations and specifically in the context of cancers that contain wild-type PI3KCA [61,62]. Its characterization as an oncogenic driver adds to the rationale for targeting PIK3CB therapeutically. PIK3C2B also belongs to the PI3K family. It contains a lipid kinase catalytic domain as well as a C-terminal C2 domain, a characteristic of class II PI3-kinases. PIK3C2B has been implicated in epithelial-to-mesenchymal transition, and insensitivity to EGFR inhibitors [63]. While PIK3CA mutations are frequent in head and neck squamous cell carcinomas, mutations involving PIK3CB or PIK3C2B are relatively rare [56]. Yet, the significant co-expression we found between the ErbBs and both PIK3CB and PIK3C2B, and the demonstrated link between constitutive Akt activation and resistance to EGFR inhibition in head and neck cancers [54,64,65], suggests that PI3K inhibitors could have added therapeutic effects also in PIK3CA non-mutant tumors (Figure 2b).

Overexpression of protein kinase C epsilon (PRKCE), a phorbol ester receptor, is a hallmark of multiple cancers and has been widely implicated in malignant transformation, tumor aggressiveness, and metastasis [66]. EGFR activation induces PRKCE monoubiquitylation at Lys321 mediated by RINCK1 ubiquitin ligase [67]. Higher levels of PRKCE were found to correlate with an increase in disease recurrence and a decrease in overall survival in head and neck cancer [68]. Targeted disruption of PRKCE has been shown to reduce cell invasion and motility through inactivation of RhoA and RhoC GTPases in head and neck squamous cell carcinoma [69]. Multi-protein kinase inhibitors can be used to target protein kinase C family members (Figure 2b).

Chromodomain helicase DNA binding protein 4 (CHD4) belongs to the SNF2/RAD54 helicase family and represents the core component of the nucleosome remodeling and deacetylase (NuRD) complex, thus playing an important role in epigenetic regulation. CHD4 was found to be commonly mutated across various cancer types [70]. CHD4 augments ErbB2-mediated signaling cascades [71]. The biologic role of CHD4 in head and neck cancers remains elusive, but its identification as an oncogenic and cancer stem cell element associated with metastatic potential may implicate it as a novel therapeutic target for the treatment of various cancers [72,73,74]. A novel causative role was discovered for CHD4 as a major predictor of sensitivity to HDAC inhibitors [75]. Given that HDAC inhibitors are currently being tested in clinical trials for head and neck squamous cell carcinomas and the demonstrated synergism when combined with EGFR inhibitors [47,48], CHD4 could constitute a clinically relevant response biomarker (Figure 2b).

AT-Rich Interaction Domain 1A (ARID1A) is part of the chromatin remodeling complex SWI/SNF, which has helicase and ATPase activities and regulates the transcription of certain genes by altering the chromatin structure around those genes. Recent data suggest that ARID1A participates in tumor progression through its effects on the control of cell cycle, modulation of cellular functions such as epithelial-to-mesenchymal transition, and regulation of various signaling pathways [76]. Epigenetic driver mutations in ARID1A occur in human cancers and promote cancer development [77,78,79]. Importantly, these mutations can shape cancer immune phenotype and immunotherapy [80,81]. The immunosuppressive tumor microenvironment plays an essential role in the treatment of head and neck cancers. ARID1A mutations have been described as potential predictors of immune checkpoint inhibitor efficacy in head and neck squamous cell carcinoma [82]. Given its immunomodulating effects and the increasingly important link between EGFR inhibitory therapies and tumor immune microenvironment [83], ARID1A changes may provide clues for optimizing immune checkpoint inhibitor therapy in head and neck cancers (Figure 2b).

Polybromo 1 (PBRM1) also represents a subunit of ATP-dependent chromatin-remodeling complexes. PBRM1 is frequently mutated in renal cell carcinoma and drives carcinogenesis [84]. PBRM1 loss defines a non-immunogenic tumor phenotype associated with immune checkpoint inhibitor resistance [85,86,87,88]. Moreover, loss-of-function PBRM1 mutations attenuate the effects of EGFR inhibition in part by sustaining Akt signaling [89], implying a role of PBRM1 not only as a biomarker of immune checkpoint inhibitor response, but also as a potential modifier of EGFR dependency.While these genes represent potential action points to modulate an ErbB-driven co-regulon in head and neck squamous cell carcinoma, the simultaneous overexpression of multiple ErbB receptors in most cases [15] suggests that concurrent modulation of multiple ErbB family members could be biologically and clinically meaningful. This notion is supported by the observation that agents targeting EGFR produce significant therapeutic benefit [12,16,17]; however, overall effect has been modest owing to resistance involving the ErbB pathway. Given substantial redundancy of signaling circuitries involving the ErbBs and the documented signaling potency emanating from ErbB receptor combinations [25,26,27,28,29,30], such co-targeting strategies involving several ErbB members could have potential therapeutic merit.

While having identified a target-rich environment for multimodal therapeutic approaches in ErbB-driven head and neck squamous cell carcinomas, the results of our study are preliminary and require validation. Target validation will need to include confirmation that our identified candidate genes, in fact, have key roles in head and neck cancer pathogenesis and that their pharmacologic modulation is feasible (‘druggability’). Such validation must progress from laboratory exploration of drug synergisms between ErbB-targeted therapies and the drug classes that we have identified here, to the subsequent initiation of efforts to test such synergisms in preclinical and, eventually, early clinical trials. This transition must include target assessment aspects such as ‘assayability’ of the potential for target modulation to achieve differentiation from established therapies. As studies on promising gene-drug interactions and drug synergisms advance towards clinical trials in patients, additional aspects become critical, including pharmacokinetic (PK)-pharmacodynamic (PD) analyses and drug/target-related safety issues, and gauging those against the extent of unmet medical need in distinct head and neck cancer populations, e.g., human papillomavirus (HPV)-positive vs. -negative subpopulations. HPV-positive tumors demonstrate a distinct genetic makeup than HPV-negative tumors [13] that might impact the ErbB co-regulon identified in this study. Therefore, it will need to be critically evaluated whether effect sizes of modulating our identified gene-drug interactions are of a magnitude that would be clinically meaningful in subsets of patients with differing HPV status. Such studies will be key to ultimately devising new lines of combinatorial treatment strategies for ErbB-dependent head and neck cancers that incorporate the findings of our study.

## Supplementary Materials

The following supporting information can be downloaded at: https://www.mdpi.com/article/10.3390/ijms232113497/s1.
